# Patterns of regional gray matter loss at different stages of schizophrenia: A multisite, cross-sectional VBM study in first-episode and chronic illness^☆^

**DOI:** 10.1016/j.nicl.2016.06.002

**Published:** 2016-06-03

**Authors:** Ulysses S. Torres, Fabio L.S. Duran, Maristela S. Schaufelberger, José A.S. Crippa, Mario R. Louzã, Paulo C. Sallet, Caroline Y.O. Kanegusuku, Helio Elkis, Wagner F. Gattaz, Débora P. Bassitt, Antonio W. Zuardi, Jaime Eduardo C. Hallak, Claudia C. Leite, Claudio C. Castro, Antonio Carlos Santos, Robin M. Murray, Geraldo F. Busatto

**Affiliations:** a Post-Graduation Program in Radiology, Institute of Radiology (INRAD), Faculty of Medicine, University of São Paulo, Brazil; b Laboratory of Psychiatric Neuroimaging (LIM-21), Department and Institute of Psychiatry, Faculty of Medicine, University of São Paulo, Brazil; c Center for Interdisciplinary Research on Applied Neurosciences (NAPNA), University of São Paulo, Brazil; d Department of Neuroscience and Behaviour, School of Medicine of Ribeirão Preto, University of São Paulo, Ribeirão Preto, Brazil; e Department and Institute of Psychiatry, University of Sao Paulo Medical School, Brazil; f Department of Diagnostic Imaging, Heart Institute (InCor), Faculty of Medicine, University of São Paulo, Brazil; g Laboratory of Neuroscience (LIM 27), Department and Institute of Psychiatry, Faculty of Medicine, University of São Paulo, Brazil; h Department of Internal Medicine – Radiology Division, School of Medicine of Ribeirão Preto, University of São Paulo, Ribeirão Preto, Brazil; i Department of Psychosis Studies, Institute of Psychiatry, King's College London, UK

**Keywords:** Voxel-based morphometry, MRI, Schizophrenia

## Abstract

Background: Structural brain abnormalities in schizophrenia have been repeatedly demonstrated in magnetic resonance imaging (MRI) studies, but it remains unclear whether these are static or progressive in nature. While longitudinal MRI studies have been traditionally used to assess the issue of progression of brain abnormalities in schizophrenia, information from cross-sectional neuroimaging studies directly comparing first-episode and chronic schizophrenia patients to healthy controls may also be useful to further clarify this issue. With the recent interest in multisite mega-analyses combining structural MRI data from multiple centers aiming at increased statistical power, the present multisite voxel-based morphometry (VBM) study was carried out to examine patterns of brain structural changes according to the different stages of illness and to ascertain which (if any) of such structural abnormalities would be specifically correlated to potential clinical moderators, including cumulative exposure to antipsychotics, age of onset, illness duration and overall illness severity.

Methods: We gathered a large sample of schizophrenia patients (161, being 99 chronic and 62 first-episode) and controls (151) from four previous morphometric MRI studies (1.5 T) carried out in the same geographical region of Brazil. Image processing and analyses were conducted using Statistical Parametric Mapping (SPM8) software with the diffeomorphic anatomical registration through exponentiated Lie algebra (DARTEL) algorithm. Group effects on regional gray matter (GM) volumes were investigated through whole-brain voxel-wise comparisons using General Linear Model Analysis of Co-variance (ANCOVA), always including total GM volume, scan protocol, age and gender as nuisance variables. Finally, correlation analyses were performed between the aforementioned clinical moderators and regional and global brain volumes.

Results: First-episode schizophrenia subjects displayed subtle volumetric deficits relative to controls in a circumscribed brain regional network identified only in small volume-corrected (SVC) analyses (p < 0.05, FWE-corrected), including the insula, temporolimbic structures and striatum. Chronic schizophrenia patients, on the other hand, demonstrated an extensive pattern of regional GM volume decreases relative to controls, involving bilateral superior, inferior and orbital frontal cortices, right middle frontal cortex, bilateral anterior cingulate cortices, bilateral insulae and right superior and middle temporal cortices (p < 0.05, FWE-corrected over the whole brain). GM volumes in several of those brain regions were directly correlated with age of disease onset on SVC analyses for conjoined (first-episode and chronic) schizophrenia groups. There were also widespread foci of significant negative correlation between duration of illness and relative GM volumes, but such findings remained significant only for the right dorsolateral prefrontal cortex after accounting for the influence of age of disease onset. Finally, significant negative correlations were detected between life-time cumulative exposure to antipsychotics and total GM and white matter volumes in schizophrenia patients, but no significant relationship was found between indices of antipsychotic usage and relative GM volume in any specific brain region.

Conclusion: The above data indicate that brain changes associated with the diagnosis of schizophrenia are more widespread in chronic schizophrenia compared to first-episode patients. Our findings also suggest that relative GM volume deficits may be greater in (presumably more severe) cases with earlier age of onset, as well as varying as a function of illness duration in specific frontal brain regions. Finally, our results highlight the potentially complex effects of the continued use of antipsychotic drugs on structural brain abnormalities in schizophrenia, as we found that cumulative doses of antipsychotics affected brain volumes globally rather than selectively on frontal-temporal regions.

## Highlights

• Structural brain changes are more widespread in chronic than first-episode schizophrenia.
• Regional GM deficits may be greater in cases with earlier age of onset.
• Illness duration seems to impact in some specific frontal structural brain changes.
• Antipsychotics seem to affect brain volumes globally rather than regionally.

## 1 Introduction

Structural brain abnormalities have been repeatedly demonstrated in groups of patients with schizophrenia compared to healthy controls in magnetic resonance imaging (MRI) studies, with some of the findings being observed at (or even before) the onset of illness (Steen et al., 2006, Vita et al., 2012, Ellison-Wright et al., 2008, Shenton et al., 2001, Fusar-Poli et al., 2011, Smieskova et al., 2010). However, it is not clear whether such structural brain changes are static or progressive in nature (Vita et al., 2012, van Haren et al., 2008, DeLisi, 2008, Weinberger and McClure, 2002, Chan et al., 2011, Zipursky et al., 2013, Olabi et al., 2011).

Although longitudinal MRI studies have been traditionally used to address the question of progression of brain abnormalities in schizophrenia, information from cross-sectional neuroimaging studies may also provide useful contributions to further clarify this issue (Pantelis et al., 2005). Cross-sectional MRI comparisons of patients with first-episode and chronic schizophrenia are of interest because if there are more widespread brain changes in chronic than in first-episode psychosis, then this could imply a progression of brain anatomical changes after symptom onset (Ellison-Wright et al., 2008). Moreover, if structural brain changes are less widespread in first-episode schizophrenia, then the foci of such abnormalities could be seen as the critical brain regions of neuropathology from which continuing abnormalities may evolve to further, more widespread anatomical changes in chronic schizophrenia (Ellison-Wright et al., 2008). However, such cross-sectional approach has hitherto been applied primarily in meta-analytic investigations that indirectly compared groups of MRI studies on first-episode versus groups of studies on chronic schizophrenia (Vita et al., 2012, Ellison-Wright et al., 2008, Chan et al., 2011, Olabi et al., 2011, Honea et al., 2005). Conversely, original cross-sectional MRI studies directly comparing first-episode and chronic schizophrenia patients to controls selected from the same geographical area have been very scarce to date (Meisenzahl et al., 2008). Not infrequently, cross-sectional MRI studies actually pool together both chronic and first-episode patients (Meda et al., 2008, Antonova et al., 2005, Honea et al., 2008), do not explicitly report the duration of illness (Garcia-Marti et al., 2008, Moorhead et al., 2004, Tregellas et al., 2007), or even do not apply strict criteria to define these patient groups in regard to the stage of illness (Keshavan & Schooler, 1992).

Recently, there has been growing interest in multisite mega-analyses combining structural MRI data from multiple centers through a uniform procedure of preprocessing and analysis, aiming at increased sensitivity and statistical power to detect group differences and investigate within-group correlations with clinical variables (de Wit et al., 2014, Gupta et al., 2014, Jednorog et al., 2015, Mueller et al., 2005, Segall et al., 2009, Turner et al., 2012). Three of these multisite MRI studies have evaluated subjects with schizophrenia applying the voxel-based morphometry (VBM) approach (Gupta et al., 2014, Segall et al., 2009, Turner et al., 2012), whereby differences in gray matter (GM) volumes between schizophrenia patients and controls were investigated on a voxel-by-voxel basis across the entire brain. Also, the increased statistical power arising from VBM studies with large samples may help to disentangle to what extent progression of brain changes may be a consequence of an ongoing pathophysiological process in schizophrenia or a result of potential clinical moderators such as antipsychotics usage, for example. This is relevant since recent studies have suggested that antipsychotics, the cornerstone of the treatment of schizophrenia, may be significantly associated to some of the progressive neuroanatomical changes previously reported in some MRI studies of schizophrenia, even after accounting for other potential moderators such as disease duration, illness severity and substance abuse (Ho et al., 2011, Fusar-Poli et al., 2013, Torres et al., 2013). Moreover, VBM studies using large schizophrenia and control samples may also afford statistical power to evaluate the potential influence of other clinical moderators on GM volumes, such as duration of illness and the overall severity of schizophrenia clinical features.

In the present cross-sectional, multisite VBM study, we sought to replicate findings of brain structural changes in schizophrenia reported in previous large-sized VBM investigations (Vita et al., 2012, Ellison-Wright et al., 2008, Chan et al., 2011, Olabi et al., 2011, Honea et al., 2005) by comparing groups of first-episode and chronic patients relative to demographically matched controls, all recruited in the state of São Paulo, Brazil. We hypothesized that chronic schizophrenia patients would have a more spatially widespread pattern of brain volume abnormalities than first-episode schizophrenia patients. Also, we aimed to ascertain which (if any) of such structural brain changes would be specifically correlated to potential clinical moderators including cumulative exposure to antipsychotics, age of onset, illness duration and overall illness severity.

## 2 Methods

### 2.1 Subjects

The present investigation gathered a large sample of schizophrenia patients and controls from four previous morphometric MRI studies carried out in medical institutions of two cities located in the same geographical region of Brazil (São Paulo State), namely the University of São Paulo Medical School in São Paulo City, and the University of São Paulo Medical School in Ribeirão Preto City. Such MRI studies had been carried out to address different research questions and their separate results have been published elsewhere (Bassitt et al., 2007, Sallet et al., 2003, Schaufelberger et al., 2007, de Souza Crippa et al., 2006). All participants provided written informed consent at the time of the original MRI studies, and permission for this multisite data analysis was obtained from the ethical review board at University of São Paulo Medical School. We included a total of 312 non-overlapping subjects, being 161 schizophrenia patients (99 chronic, 62 first-episode) and 151 controls who underwent 1.5 T structural T1-weighted MRI scanning. In studies of this kind, carried out with the primary aim to dichotomize schizophrenia samples regarding illness duration, great care must be taken to assure that chronic schizophrenia samples have strictly distinct median duration of disease in comparison to their counterpart, i.e., the first-episode sample. Therefore, when selecting studies for this mega-analysis we adopted a rigid criterion to define illness chronicity, as applied elsewhere (Chan et al., 2011): median illness duration could not be less than 5 years in each study involving chronic schizophrenia patients (Bassitt et al., 2007, Sallet et al., 2003, de Souza Crippa et al., 2006); similarly, the selected first-episode schizophrenia subjects (Schaufelberger et al., 2007) had a median duration of illness of less than 1 year (Chan et al., 2011).

Chronic schizophrenia patients were recruited at local outpatient clinics at the Institute of Psychiatry of University of São Paulo (Bassitt et al., 2007, Sallet et al., 2003) and the University Hospital of the Faculty of Medicine of Ribeirão Preto (de Souza Crippa et al., 2006). Differently, first-episode schizophrenia patients (Schaufelberger et al., 2007) were drawn from a sample of people with first-episode psychosis identified for an epidemiological study of the incidence of psychotic disorders in São Paulo (Menezes et al., 2007); people presenting with a psychotic illness and who made contact for the first time with the mental healthcare services for that region (a circumscribed geographical area of São Paulo comprising a total of approximately 900,000 inhabitants) were recruited by active surveillance.

Inclusion criteria for schizophrenia subjects were: diagnosis of schizophrenia according to DSM-IV criteria on the basis of interviews with the Structured Clinical Interview for DSM-IV Axis I Disorders for all studies (First et al., 1996); and an age at the time of MRI scanning ranging between 18 to 50 years in three studies (Bassitt et al., 2007, Schaufelberger et al., 2007, de Souza Crippa et al., 2006), and 18 to 60 years in the remaining study (Sallet et al., 2003). People with psychotic disorders due to a general medical condition or substance-induced psychosis were excluded.

In three of the MRI studies (Bassitt et al., 2007, Sallet et al., 2003, de Souza Crippa et al., 2006), healthy controls were recruited from the local community through local advertisements or invited by word of mouth, which in one study also included members from the staff at the Institute of Psychiatry at the University of São Paulo Medical School in São Paulo city (Bassitt et al., 2007). For the remaining MRI study (the study on first-episode patients) (Schaufelberger et al., 2007), an epidemiological approach was applied for the recruitment of controls, randomly selecting a large group without psychosis from the same geographical area, comprised by next-door neighbors from first-episode patients; such population-based approach was used in that study in order to minimize the chance of selection bias (32).

Exclusion criteria specific for the control group were personal history of other Axis I disorders (as determined by interviews using the SCID) (Bassitt et al., 2007, Sallet et al., 2003, de Souza Crippa et al., 2006) or psychosis (Bassitt et al., 2007, Sallet et al., 2003, Schaufelberger et al., 2007, de Souza Crippa et al., 2006). Additional exclusion criteria for both groups were: history of head injury (Bassitt et al., 2007, Sallet et al., 2003, Schaufelberger et al., 2007, de Souza Crippa et al., 2006); presence of neurological disorders or any organic disorders that could affect the central nervous system (Bassitt et al., 2007, Sallet et al., 2003, Schaufelberger et al., 2007, de Souza Crippa et al., 2006); previous treatment with steroid medication (Sallet et al., 2003); contraindications for MRI scanning (Bassitt et al., 2007, Sallet et al., 2003, Schaufelberger et al., 2007, de Souza Crippa et al., 2006); major medical illnesses (de Souza Crippa et al., 2006); and substance abuse (Sallet et al., 2003, de Souza Crippa et al., 2006).

Detailed sociodemographic and clinical data were retrieved from schizophrenia patients of each site. Although different scales for psychopathology severity were originally published in each study (Bassitt et al., 2007, Sallet et al., 2003, Schaufelberger et al., 2007, de Souza Crippa et al., 2006), scores of the Positive and Negative Syndrome Scale (PANSS) (Kay et al., 1987) were commonly available in most of the databases (Bassitt et al., 2007, Sallet et al., 2003, Schaufelberger et al., 2007), except for one (de Souza Crippa et al., 2006); therefore, for the present mega-analysis, symptom severity was rated using the PANSS, using only the three samples that had these data available.

We also collected data on antipsychotics usage (typical or atypical drugs, duration of exposure, dose at the time of MRI scanning and lifetime cumulative exposure). Given that many schizophrenia patients are maintained on antipsychotic treatment for long periods, it is useful to quantitatively assess cumulative medication exposure, especially because recent studies have highlighted the potential association between structural brain changes and use of antipsychotics in schizophrenia (Ho et al., 2011, Torres et al., 2013, Moncrieff and Leo, 2010, Smieskova et al., 2009, Navari and Dazzan, 2009). In order to calculate lifetime cumulative antipsychotic exposure, we first converted antipsychotic doses to chlorpromazine (CPZ) milligram equivalent units (Andreasen et al., 2010). We subsequently calculated, for each patient, total cumulative exposure expressed in dose-years (Andreasen et al., 2010). In cases of chronic antipsychotics exposure, it is worth mentioning that antipsychotic treatment is naturalistic and patients usually receive a standard treatment at discretion of treating psychiatrist (Ho et al., 2011). Thus, in large, multisite studies like ours, by using retrospective methods (chart reviews), available data on antipsychotics may be heterogeneous and somewhat limited, making it difficult to obtain measurements of lifetime antipsychotic exposure with accuracy (Ho et al., 2011). Therefore, we derived cumulative antipsychotic exposure using a product of defined daily dose and duration of illness as determined from patients' charts. This index can be taken as an approximate measure of lifetime antipsychotic exposure (Palaniyappan et al., 2014).

### 2.2 MRI acquisition and image processing

High-resolution T1-weighted images were acquired in four different sites of the two medical institutions, with imaging parameters as summarized in Table 1. Prior to data processing, all scans successfully passed a quality assessment protocol, based on visual inspection for gross structural abnormalities, artifacts, or poor image quality that could potentially hamper image segmentation. Imaging data processing and analysis were carried out using VBM methods based on the Statistical Parametric Mapping 8 software package (SPM8; Wellcome Trust Centre for Neuroimaging; http://www.fil.ion.ucl.ac.uk/spm/software/spm8/) run in MATLAB (Mathworks, USA). Briefly, following manual reorientation in order to place the anterior commissure at the origin of the three-dimensional Montreal Neurological Institute (MNI) coordinate system, T1-W images were segmented into tissue classes of GM, white matter (WM), and cerebrospinal fluid (CSF) by using the standard unified segmentation model in SPM8 (Ashburner & Friston, 2005), which makes use of tissue probability maps reflecting the prior probability of a given voxel belonging to any tissue class (Ashburner & Friston, 2005). The Diffeomorphic Anatomical Registration Through Exponentiated Lie Algebra (DARTEL) algorithm (Ashburner, 2007) was employed to create a study-specific template for spatial normalization of the segmented images of each subject.

The resulting flow fields created by DARTEL were used to generate GM and WM images of each individual; such images were spatially normalized in the MNI space, modulated, resliced (1.0-mm isotropic voxels), and smoothed (8-mm full-width at half maximum Gaussian kernel). In order to compensate for the effects of spatial normalization, GM and WM normalized images are modulated by multiplying each voxel value by its relative volume before and after warping, which allows obtaining relative volume for each voxel; the maps so produced are referred to as images of GM and WM volumes in order to distinguish them from the images of “concentration” or “density” that result if the modulation stage is omitted (Segall et al., 2009, Sundram et al., 2010). Therefore, volumes of GM, WM and CSF were calculated by the MATLAB get_totals script (http://www.cs.ucl.ac.uk/staff/g.ridgway/vbm/get_totals.m) implemented for SPM8 on the native space gray, white, and cerebrospinal fluid segmentations for each subject. Similarly, to compute total intracranial volume (TIV) using SPM's unified segmentation and spatial normalization procedure, probabilistic tissue class images can be integrated, with the estimated TIV simply being the sum of GM, WM and CSF volumes (Ansell et al., 2012, Benedetti et al., 2010, Malone et al., 2015), as herein adopted. Total brain volume (TBV) was estimated by summing GM and WM volumes (Nordenskjöld et al., 2013). However, tissue prior probability templates used in SPM are based on averaging multiple automatically segmented images in standard space, with no certainty that the sum of these three compartments will be exactly consistent with accepted definitions of TIV (Malone et al., 2015). For instance, such automated method of TIV estimation in SPM8 was found to overestimate TIV by 20.86% as compared to manual segmentation (Nordenskjöld et al., 2013), these errors being shown to impact the ability to detect differences in hippocampal volume, for example (Nordenskjöld et al., 2013). It is expected that improved new segmentation algorithms incorporated into SPM8 and SPM12 can produce more accurate TIV results, despite current evidence for this are yet scarce (Malone et al., 2015).

Finally, in order to ensure that all images were segmented adequately, subject outlier detection was performed within and across the four sites using a Pearson correlation, by comparing the degree to which subjects were related to the average smoothed GM map. Outliers were defined as subjects deviating more than 2% from the mean GM volumes, according to criteria defined elsewhere (Segall et al., 2009); no outlier was identified.

### 2.3 Statistical analysis

Group differences in demographic characteristics were assessed with IBM SPSS Statistics, version 21 (IBM, Armonk, USA). The significance threshold was set at 0.05, two-tailed; continuous variables were analyzed through the *t* test and categorical variables through Pearson's χ^2^ test.

Regarding the VBM analyses, group effects on regional GM volume were investigated through whole-brain voxel-wise comparisons of the preprocessed GM images of schizophrenia patients and controls by using General Linear Model (GLM) Analysis of Co-variance (ANCOVA), which always included total GM volume, scan protocol, age and gender as nuisance variables (hereafter defined as “standard covariance”). Moreover, within-group correlations were performed between regional brain volumes and potential clinical moderators [psychopathology severity (as assessed by PANSS total score, after excluding subjects of one study with unavailable data (de Souza Crippa et al., 2006)), life-time cumulative exposure to antipsychotics (as assessed by estimation of dose-years), age of onset and duration of illness], always correcting for the effects of the remainder potential clinical moderators, in addition to the standard covariance.

It has been previously described that variations in the choice of method for adjusting VBM-based regional GM volume indices for global GM (total GM, TGM, i.e., the sum of GM across all voxels) may significantly affect results when making inferences about regional brain volume changes (Peelle et al., 2012). Accordingly, we conducted additional VBM analyses using three alternative methods of covariation for global GM, as described elsewhere (Peelle et al., 2012). First, for each individual, we calculated TGM as the sum of GM across brain regions using unregistered, unsmoothed MRI datasets (Peelle et al., 2012). Subsequently, using the aforementioned definition of TIV, the following VBM analyses were performed: *no adjusting for TGM*, with and without total intracranial volume (TIV) being considered; *global scaling*, by proportionally scaling each participant's GM image by the TGM from that image (i.e., each participant's smoothed normalized GM image was divided by the TGM for that participant), with and without TIV being considered; and *local covariation*, by including each participant's TGM as an additional covariate of no interest in an ANCOVA, with and without TIV being considered (Peelle et al., 2012). Results of such analyses were subsequently compared to those of our conventional approach in order to verify if the neuroanatomical pattern and level of extension of regional GM changes were substantially affected in this study according to the method of global adjusting.

For all VBM analyses, the statistics for each voxel of the whole GM compartment were transformed to Z scores and displayed as statistical parametric maps (SPMs) in standard MNI space at an initial threshold probability of Z > 3.09 (p < 0.001, uncorrected), with cluster size threshold of 20 contiguous voxels (Uwatoko et al., 2015, Fusar-Poli et al., 2012). When examining the voxelwise statistical maps, we did not consider significant findings with spatial patterns primarily around the edges of the brain, in regions subject to artifact of partial volume and deskulling problems (Honea et al., 2008, Gupta et al., 2014). Statistical inference was conducted in two steps. Initially, we used a strict statistical threshold of p < 0.05, familywise error (FWE)-corrected for multiple comparisons over the whole brain. Second, we applied small volume correction (SVC) over the following brain regions predicted a priori to show abnormalities in schizophrenia patients, based on recent meta-analyses and mega-analyses of structural MRI studies on schizophrenia (Chan et al., 2011, Bora et al., 2011, Kuhn and Gallinat, 2013): insula, dorsolateral prefrontal cortex, superior temporal gyrus, amygdala, hippocampus/parahippocampal gyrus, anterior cingulate cortex, thalamus and striatum. Such brain regions were spatially delimited by applying normalized volumes onto the SPMs, based on the anatomical volumes of interest that are available within the automatic anatomical labeling SPM toolbox. Such flexible statistical analyses were conducted using a FWE-corrected threshold of p < 0.05 over the volume of the SVC-based region, also with cluster size threshold of 20 contiguous voxels. The SVC approach allows hypothesis-driven analyses to be performed with correction for multiple comparisons specifically in the brain region of interest rather than using a stricter correction for the whole brain (Schaufelberger et al., 2007).

Finally, for the conjoined group (chronic + first-episode), as well as for the chronic and first-episode groups separately, we also performed bivariate correlation analyses (Pearson product-moment correlation, two-tailed, p < 0.05) between global volume values (GM, WM, CSF, TBV and TIV) and the following clinical moderators: psychopathology severity [as assessed by PANSS total score, after excluding subjects of one study with unavailable data (de Souza Crippa et al., 2006)], life-time cumulative exposure to antipsychotics (as assessed by estimation of dose-years), duration of illness, and age of onset. The effects of the remainder clinical variables on the results of correlation analyses between a given clinical moderator and global brain volumes were also accounted for through partial correlation analyses (two-tailed, p < 0.05).

In order to give a general overview of the main VBM findings of the study, regardless of as to whether they were significant in the whole-brain exploratory analyses (at strict statistical significance levels) or in the (more flexible) SVC-based analyses, all figures were constructed based on statistical parametric maps thresholded at p < 0.001 (uncorrected for multiple comparisons) with a minimum cluster extent of 10 voxels, as employed elsewhere (de Wit et al., 2014).

## 3 Results

### 3.1 Demographic and clinical variables

Sociodemographic and clinical data are listed in Table 2. Patients and controls did not differ in terms of age, but there was a significantly higher male predominance among schizophrenia patients in comparison to controls, as well as significantly fewer years of education in schizophrenia patients (Table 1). Chronic schizophrenia patients did not differ from first-episode schizophrenia patients regarding gender, but they were significantly older than first episode schizophrenia patients, had higher number of years of education, a lower age of illness onset and higher PANSS scores (Table 2). Most chronic schizophrenia patients were on atypical antipsychotics usage, while typicals were the most common antipsychotic class among first episode schizophrenia patients, this difference being statistically significant (Table 2). As would be expected, chronic schizophrenia patients had higher life-time cumulative exposure to antipsychotics.

### 3.2 Global volume analyses

Patients and controls did not differ in terms of total GM or WM volumes, TBV and TIV (Table 3). Patients with schizophrenia had higher CSF volumes than controls (p < 0.0001). Chronic schizophrenia patients did not differ from first-episode patients regarding total GM or WM volumes, but CSF volumes were larger (p < 0.0001) among chronic in comparison to first-episode patients (Table 3). However, this difference did not remain statistically significant when the effect of age of onset was accounted for (Table 3).

Bivariate correlation analyses revealed significant negative correlations between life-time cumulative exposure to antipsychotics and global volumes for both the conjoined group [(total GM, r = − 0.244, p = 0.002); (total WM, r = − 0.177, p = 0.025); (TBV, r = − 0.218, p = 0.005); (TIV, r = − 0.157, p = 0.047)] and for the chronic schizophrenia group [(total GM, r = − 0.372, p = 0.000); (total WM, r = − 0.208, p = 0.039); (TBV, r = − 0.305, p = 0.002); (TIV, r = − 0.271, p = 0.007)]. When these analyses for antipsychotic exposure were repeated in the chronic schizophrenia group with covariance for disease duration, both the negative correlations with total GM (r = − 0.207, p = 0.044) and TBV (r = − 0.202, p = 0.044) retained statistical significance, while the negative correlations with total WM (r = − 0.188, p = 0.067) and TIV (r = − 0.186, p = 0.071) remained as significant trends. Repeated analyses for the conjoined four groups with covariance for disease duration indicated trend significance for the negative correlation between total GM and antipsychotic exposure (r = − 0.143, p = 0.075).

For the conjoined group only, a significant positive correlation was found between CSF volumes and duration of disease (r = 0.237; p = 0.002). However, this finding lost its statistical significance after covariance for antipsychotic exposure and age of disease onset (r = 0.027, p = 0.741).

No significant correlations between severity of disease and global volumes were found for any group.

### 3.3 Regional brain volume analyses: VBM findings of between-group comparisons

**Fig. 1:**
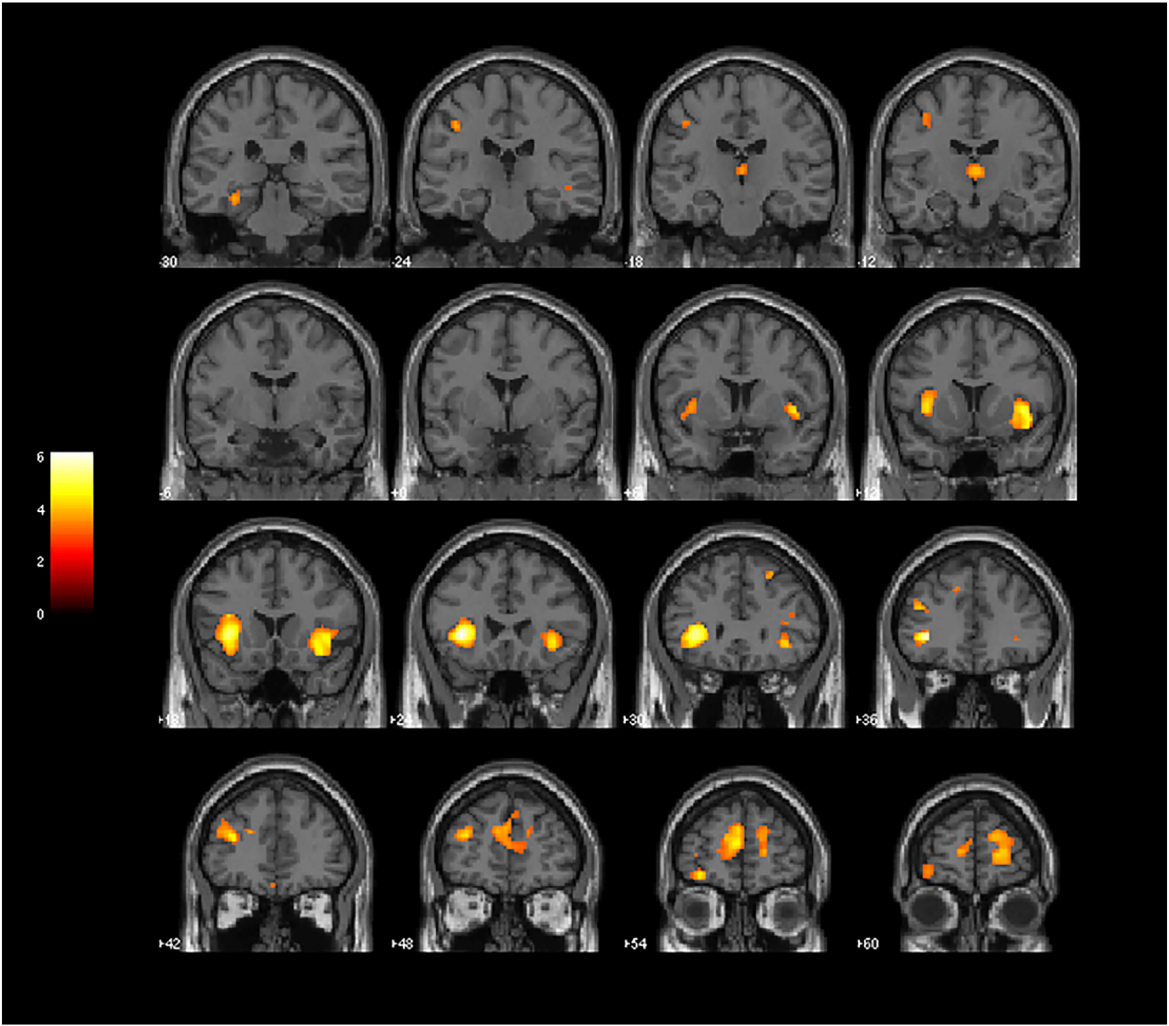
Results of VBM analyses showing GM reductions occurring in the combined group of chronic and first-episode schizophrenia patients (n = 161) versus controls (n = 151). Brain slices are in coronal planes, and the right hemisphere of the brain is on the right. Statistical parametric maps were thresholded at p < 0.001 uncorrected for multiple comparisons with a minimum cluster extent of 10 voxels. Findings are highlighted in the bilateral insulae, right striatum and left inferior frontal gyrus (significant at p < 0.05, FWE-corrected over the whole brain), as well as in the bilateral dorsolateral prefrontal cortices, bilateral anterior cingulate cortices, bilateral thalami and left hippocampus/parahippocampal gyrus (significant in SVC analyses at p < 0.05, FWE-corrected) (see Table 4 for statistical and localization details).

The VBM analysis of main effect of diagnosis, under the strict threshold of p < 0.05 (FWE-corrected) and minimum cluster extent of 20 voxels over the whole brain, revealed a pattern of cortical volume deficits restricted to the bilateral insulae, right striatum and left inferior frontal gyrus when contrasting all schizophrenia patients versus healthy controls (Fig. 1, Table 4). Small volume-corrected analyses revealed reduced GM volumes in schizophrenia patients relative to controls in the following additional brain regions predicted a priori to show abnormalities: bilateral dorsolateral prefrontal cortices, bilateral anterior cingulate cortices, bilateral thalami and left hippocampus/parahippocampal gyrus. The voxel extent of such findings and corresponding Z-scores are provided in Table 4.

**Fig. 2:**
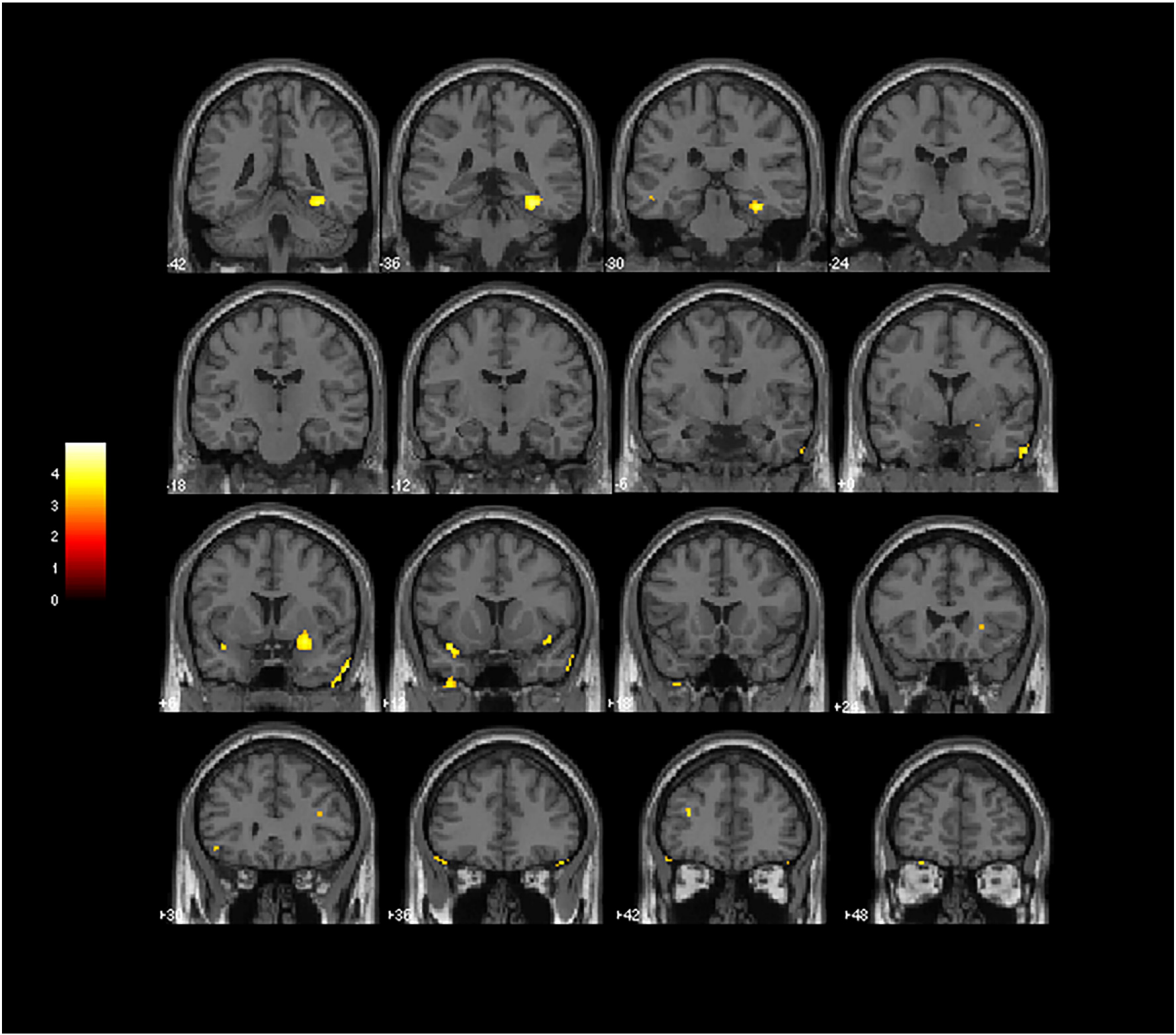
Results of VBM analyses showing GM reductions occurring in first-episode schizophrenia patients (n = 62) versus controls (n = 151) identified in SVC analyses at p < 0.05, FWE-corrected. Brain slices are in coronal planes, highlighting findings in the bilateral insulae, right amygdala, right striatum and right hippocampus/parahippocampal gyrus. The right hemisphere of the brain is on the right. Statistical parametric maps were thresholded at p < 0.001 uncorrected for multiple comparisons with a minimum cluster extent of 10 voxels.

There were no regional GM volume deficits in the first-episode schizophrenia sample relative to controls at FWE-corrected analyses over the whole brain. Small volume-corrected analyses revealed reduced GM volumes in first-episode schizophrenia patients in the following brain regions predicted a priori to show abnormalities: bilateral insulae, right amygdala, right striatum and right hippocampus/parahippocampal gyrus (Fig. 2). The voxel extent of such findings and corresponding Z-scores are provided in Table 5. As a secondary analysis, we carried out a voxelwise comparison of regional WM volumes between FES subject and controls; this revealed no significant findings (at the FWE-corrected p < 0.05 level over the whole brain).

**Fig. 3:**
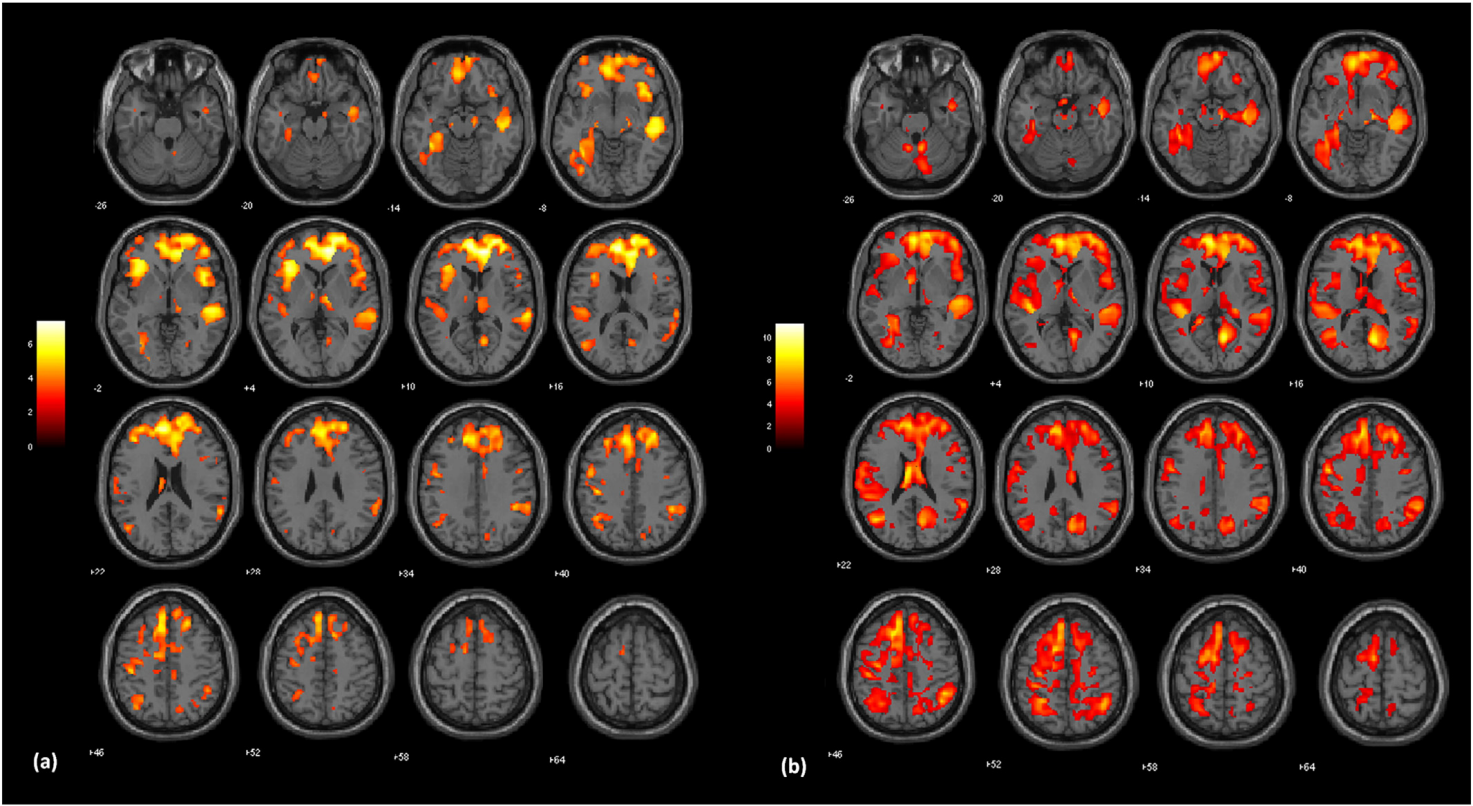
Results of VBM analyses showing GM reductions occurring in chronic schizophrenia patients (n = 99) versus controls (n = 151) (panel A), and chronic versus first-episode schizophrenia patients (n = 62), without covariation for age of onset (panel B). Brain slices are in axial planes in order to display the generalized findings of cortical and subcortical volumetric reductions in chronic patients; the right hemisphere of the brain is on the right. Statistical parametric maps were thresholded at p < 0.001 uncorrected for multiple comparisons with a minimum cluster extent of 10 voxels.

The sample of chronic schizophrenia patients presented a more extensive pattern of regional GM volume decreases relative to controls (Fig. 3a), involving the bilateral superior, inferior and orbital frontal cortices, right middle frontal cortex, bilateral anterior cingulate cortices, bilateral insulae, right superior and middle temporal cortices, left postcentral gyrus, left fusiform gyrus, right thalamus, right supramarginal gyrus, left precentral gyrus, right calcarine gyrus, left median cingulate and paracingulate gyri, and left hippocampus/parahippocampal gyrus (FWE-corrected analyses over the whole brain), as shown in Table 6. Small volume-corrected analyses revealed reduced GM volumes in the following additional brain regions predicted a priori to show abnormalities: left thalamus, right hippocampus/parahippocampal gyrus, and right superior temporal gyrus. The voxel extent of such findings and corresponding Z-scores are provided in Table 6.

In comparison to first-episode patients, chronic schizophrenia patients showed extended GM decreases (Fig. 3b) within the bilateral superior and middle temporal cortices, extending to left parahippocampal gyrus and to parietal and occipital structures (bilateral angular gyri, right supramarginal gyrus, and left inferior occipital, fusiform and lingual cortices); other brain regions involved were the left inferior frontal cortex, left superior and inferior parietal cortices, as well as left precentral and postcentral cortices, and left insula (FWE-corrected analyses over the whole brain), as shown in Table 7. Small volume-corrected analyses revealed reduced GM volumes in the following additional brain regions predicted a priori to show abnormalities: right thalamus and right amygdala. The voxel extent of such findings and corresponding Z-scores are provided in Table 7.

At the p < 0.05 (FWE-corrected) statistical threshold over the whole brain, there were no significant findings of regional GM volume increases detectable in the between-group comparisons above.

Regarding the different methods of covariation for total GM, although subtle variations in the magnitude of VBM findings were found in comparison to our standard approach, the neuroanatomical pattern of regional changes was maintained, suggesting that results of this study were not substantially affected by the method of global covariation.

### 3.4 Effects of age of disease onset on regional volume analyses (VBM)

When we repeated the above comparison between chronic and first-episode schizophrenia patients including age of onset as a confounding covariate, we detected a similar pattern of regional GM volume deficits in chronic patients relative to first-episode subjects. There were extensive GM decreases within the bilateral superior and middle temporal cortices, right superior and inferior frontal cortices, left precentral and postcentral cortices, and right occipital structures (calcarine, precuneus, cuneus and lingual cortices); other brain regions involved were the right thalamus, bilateral inferior parietal cortices and left superior parietal cortex (p < 0.05, FWE-corrected over the whole brain) (Table 8). Small volume-corrected analyses revealed reduced GM volumes in the following additional brain regions predicted a priori to show abnormalities: bilateral anterior cingulate cortices, left striatum, bilateral hippocampi/parahippocampal gyri, bilateral insulae and left thalamus. The voxel extent of such findings and corresponding Z-scores are provided in Table 8.

In the investigation of within-group correlations between regional brain volumes and age of onset, we found (only in SVC-corrected analyses) positive correlations involving the bilateral insulae and anterior cingulate and dorsolateral prefrontal cortices, the right hippocampus/parahippocampal gyrus and right superior temporal gyrus for the conjoined four groups, after accounting for the effects of life-time cumulative exposure to antipsychotics. When additionally accounting for the effects of illness severity (in the conjoined three groups with available PANSS total scores), the foci of positive correlation in the left anterior cingulate cortex, bilateral dorsolateral prefrontal cortices, left insula, right hippocampus/parahippocampal gyrus and right superior temporal gyrus all remained statistically significant. The voxel extent of these findings and their corresponding Z-scores are provided in Table 9.

### 3.5 Regional volume analyses: VBM findings of other significant within-group correlations

No significant positive or negative correlations between other potential clinical moderators (psychopathology severity, as assessed by PANSS total score; life-time cumulative exposure to antipsychotics, as assessed by estimation of dose-years; and duration of illness) and regional brain structural changes were found for any of the patient subgroups when analyses were performed under the strict threshold of p < 0.05, FWE-corrected over the whole brain.

In regard to small volume-corrected analyses, one cluster of significant negative correlation was detected in the first-episode schizophrenia group between duration of disease and GM volume of the left superior temporal gyrus (with covariance for lifetime cumulative exposure to antipsychotics, overall illness severity and age of onset). The voxel extent of this finding and its corresponding Z-score are provided in Table 10.

In the conjoined four groups, small volume-corrected analyses revealed several foci of significant negative correlation between duration of illness and GM volumes in the bilateral insulae, dorsolateral prefrontal cortices and hippocampi/parahippocampal gyri, after accounting for effects of lifetime cumulative exposure to antipsychotics (Table 10). There were additional significant negative correlations between duration of disease and GM volumes of the bilateral anterior cingulate cortices for the conjunction of the three groups with available PANSS total scores, after accounting for effects of overall illness severity and lifetime cumulative exposure to antipsychotics (Table 10). However, when these negative correlations were recalculated including age of onset as an additional confounding covariate, only a cluster in the right dorsolateral prefrontal cortex retained statistical significance, in the conjoined three groups with available PANSS total scores (Table 10).

## 4 Discussion

This large cross-sectional VBM multisite mega-analysis compared groups of schizophrenia patients (n = 161) and healthy controls (n = 151) recruited in the same geographical region. While first-episode schizophrenia subjects displayed subtle and highly circumscribed GM volume reductions in the brain relative to controls, chronic schizophrenia patients had more significant and widespread cortical GM deficits. In some brain foci, relative regional GM volumes were directly correlated with age of disease onset. Also, global brain volumes in schizophrenia were inversely related to the degree of exposure to antipsychotic drugs. While the results of our between-group comparisons provide replication to the findings of a number of previous MRI studies of schizophrenia (Vita et al., 2012, Ellison-Wright et al., 2008, Chan et al., 2011, Olabi et al., 2011, Honea et al., 2005), the relevance attributed to the potential influence of clinical moderators in our analyses is unique to date, and allowed us to unveil some novel findings.

### 4.1 Gray matter volume abnormalities associated with first-episode schizophrenia

The comparison of first-episode schizophrenia subjects against controls identified regional GM volume deficits in the former group in a circumscribed network of brain regions including the insula, temporolimbic structures (hippocampus/parahippocampal gyrus/amygdala) and striatum. Volume reductions in these brain regions have been often reported in previous VBM investigations of schizophrenia, despite some degree of variation across separate studies and meta-analyses (Ellison-Wright et al., 2008, Chan et al., 2011, Honea et al., 2005, Wright et al., 2000, Arnone et al., 2009). In our investigation, these brain volume abnormalities were subtle, being uncovered only when the more flexible SVC approach was applied.

The involvement of the insula in the pathophysiology of schizophrenia has been sometimes seen simply as an “extension” of primary frontal and temporal lobe pathology (Chan et al., 2011, Wylie and Tregellas, 2010). The relatively circumscribed findings of brain abnormalities in our first-episode schizophrenia sample, highlighting GM deficits in the insula, are not consistent with such view. Instead, the selective brain volume deficits in our first-episode schizophrenia sample, involving specifically the insula and temporolimbic structures, suggest that these foci of volume abnormalities should be seen as critical brain regions of neuropathology from which continuing abnormalities may evolve to further, widespread anatomical changes in chronic schizophrenia (Ellison-Wright et al., 2008, Smieskova et al., 2012).

The insula plays a critical role in emotional processing, integration of sensory (visual and auditory) input with the limbic system, pain perception, as well as being uniquely involved in interoception and neural representations of the self (Wylie & Tregellas, 2010). Since the latter role allows discriminating between self-generated and external information, it has been suggested that insular dysfunction may contribute to hallucinations, a cardinal feature of schizophrenia (Wylie & Tregellas, 2010). The insula has extensive connections with many areas of the cerebral cortex, and may act as an interface between the frontal and temporal lobes. Temporolimbic structures are part of a network that integrates external and internal information, mediating physiological, behavioral, and psychological responses, as well as leading to emotion-based decisions that allow for adaptation to the environment on the basis of previous experience (White et al., 2008). In addition, the medial temporal cortex is a critical component of the neural network involved in pleasure and reward (White et al., 2008). Dysfunction of temporolimbic structures is thought to critically contribute to the emergence of key clinical features of schizophrenia, including both positive and negative symptoms (White et al., 2008).

While none of the large multi-site cross-sectional VBM investigations of schizophrenia to date assessed first-episode schizophrenia patients separately, a number of single-site MRI studies have evaluated relatively large first-episode schizophrenia samples (Meisenzahl et al., 2008, Chua et al., 2007, Lui et al., 2009, Janssen et al., 2008, Job et al., 2002). In the largest single-site cross-sectional VBM study of schizophrenia to date, Meisenzahl et al. (Meisenzahl et al., 2008) found GM volume reductions in several brain regions in first-episode schizophrenia patients relative to healthy controls, affecting most significantly the perisylvian areas bilaterally, as well as the left insula, superior temporal gyrus, amygdala and hippocampus. Other bilateral GM volume reductions were found in the cingulate, orbitofrontal, dorsolateral and dorsomedial prefrontal cortices, gyrus rectus, and thalami (Meisenzahl et al., 2008). Despite the absence of significant findings involving notably the superior temporal gyrus in our study (relative reduction in the left superior temporal gyrus is the most significant finding in more than 50% of VBM studies of schizophrenia according to a meta-analysis (Honea et al., 2005)), the results of Meisenzahl et al. (Meisenzahl et al., 2008) are still partially in agreement with ours especially regarding the insular and temporo-limbic GM volume abnormalities in first-episode schizophrenia patients. In accord with our findings, other VBM studies that evaluated relatively large first-episode schizophrenia samples have also detected GM volume reductions in circumscribed regions rather than in a widespread fashion throughout the brain (Meisenzahl et al., 2008, Chua et al., 2007, Lui et al., 2009, Janssen et al., 2008, Job et al., 2002).

The first-episode schizophrenia sample included in the present study is the same evaluated previously by our group in a VBM investigation using a less optimized (SPM 2) version of the image processing software employed herein (Schaufelberger et al., 2007). The fact that the same significant results emerged in the insula and temporolimbic structures across the two investigations suggest the robustness of the findings implicating these two brain regions in first-episode schizophrenia. However, first-episode schizophrenia patients in the present investigation did not display GM volume deficits in two additional brain regions thought to be relevant to schizophrenia and which were shown to present volume abnormalities in our previous paper (Schaufelberger et al., 2007), namely the left superior temporal gyrus (as mentioned above) and the inferior lateral prefrontal cortex. Similar statistical inference approaches were applied in the two studies, and the differences in the results obtained highlight the fact that variable VBM findings may emerge when distinct spatial normalization and other routines are employed for image processing (Henley et al., 2010, Radua et al., 2014, Shen and Sterr, 2013, Callaert et al., 2014, Bergouignan et al., 2009, Tahmasebi et al., 2009). Also, a larger control group was employed in the current investigation compared to our previous publication, including a proportion of healthy individuals that were not recruited in the same neighborhood of the schizophrenia patients, as in our previous study (Schaufelberger et al., 2007). Nevertheless, it should be noted that the current SPM8-based analyses did allow detection of a significant finding involving the left superior temporal gyrus in the first-episode schizophrenia group, consisting of an inverse correlation between GM volume of this regions and disease duration before MRI scanning.

### 4.2 Greater structural brain volume deficits in chronic schizophrenia patients

Whether schizophrenia is characterized or not by progressive structural brain changes is of importance to further clarify the pathophysiology of the disorder. The neurodevelopmental hypothesis proposes that schizophrenia is a consequence of a disruption in early brain development, and that an interaction between early brain insults and later environmental factors would trigger the emergence of psychotic behavior and other overt symptoms of schizophrenia from early adulthood onwards (Weinberger, 1987, Murray, 1994). Evidence associating prenatal and perinatal complications with the diagnosis of schizophrenia later in life, absence of gliosis in the brains of schizophrenia patients as evaluated in postmortem studies, and detection of structural brain changes by MRI before illness onset, have all been taken to support the neurodevelopmental model for schizophrenia (Fusar-Poli et al., 2011, Smieskova et al., 2010, Jones et al., 1998, Falkai et al., 1999). However, the heterogeneous and sometimes deteriorating clinical course of schizophrenia suggests that neurodegenerative processes may also occur in schizophrenia, mainly in early stages after the onset of the illness (DeLisi, 2008, Lieberman et al., 1997). Evidence supporting the neurodegenerative hypothesis of schizophrenia includes findings from longitudinal neuroimaging studies showing progressive structural brain changes, such as cortical GM volume reductions and ventricular enlargement, in schizophrenia patients (Borgwardt et al., 2009). The hypothesis of neurodegeneration, however, has been controversial because the findings of different neuroimaging studies have at times been inconsistent, with some authors finding no brain volume changes over time (Whitworth et al., 2005) or even a reversal of GM decline in longitudinal MRI evaluations (Keshavan et al., 1998). Differences in image analysis techniques, duration of follow-up intervals and clinical characteristics of the schizophrenia samples recruited for each study may help to explain such heterogeneity in results of longitudinal MRI studies.

In concordance with our initial hypothesis, we detected a more widespread pattern of structural GM changes in chronic than first-episode schizophrenia patients in comparison to controls. Such pattern of results might be taken to support the notion that brain changes associated with the diagnosis of schizophrenia progress over the course of the illness. Our findings of extensive GM deficits among chronic schizophrenia patients are consistent with the results of previous meta-analyses that performed indirect comparisons between groups of first-episode and chronic schizophrenia patients (Vita et al., 2012, Ellison-Wright et al., 2008, Chan et al., 2011, Olabi et al., 2011, Honea et al., 2005). Our findings are also in agreement with the results of the previous VBM-based mega-analysis by Meisenzahl et al. (Meisenzahl et al., 2008) which applied a similar cross-sectional design to analyze MRI data from schizophrenia patients and controls. Among chronic schizophrenia patients, Meisenzahl et al. (Meisenzahl et al., 2008) found extensive GM deficits involving the insula, dorsolateral and ventrolateral prefrontal cortices, anterior cingulate cortex, temporolimbic region (including the hippocampus, amygdala and parahippocampal gyrus) and superior temporal gyrus, as well as the caudate nuclei bilaterally. Interestingly, our results demonstrate a pattern of volume abnormalities in a very similar neuroanatomical circuitry, including GM volume deficits in insular, frontal and temporolimbic brain regions in chronic schizophrenia. Such a pattern of fronto-temporolimbic volume changes in chronic schizophrenia is also in accordance with the meta-analytic seminal findings by Ellison-Wright et al. (Ellison-Wright et al., 2008). Finally, regarding the results of previous multisite mega-analyses, Segall et al. (Segall et al., 2009) evaluated 503 subjects (237 chronic patients diagnosed with schizophrenia, schizoaffective or schizophreniform disorder and 266 controls) and reported a pattern of GM reductions in the group of patients relative to controls that was most pronounced in the inferior frontal gyrus, insula, parahippocampal gyrus, superior temporal gyrus, and rectal gyrus. Such abnormalities were even more widespread in a further, larger mega-analysis evaluating chronic schizophrenia patients by Gupta et al. (Gupta et al., 2014) (1720 subjects; 784 schizophrenia patients and 936 controls), which revealed structural alterations in regions that covered most of the brain in a single cluster (i.e., whole-brain volumetric reduction in schizophrenia).

The convergent finding of large fronto-temporolimbic GM volumetric deficits in our analyses and the aforementioned studies of chronic schizophrenia subjects (Meisenzahl et al., 2008, Gupta et al., 2014, Segall et al., 2009) is consistent with the relevance attributed to these brain regions in the pathophysiology of schizophrenia (Vita et al., 2012). Fronto-temporal regions have been implicated in a range of cognitive processes affected in schizophrenia, including attention, language, working memory and other aspects of executive functioning, as well as with the expression of different clinical features of schizophrenia (Cobia et al., 2012). The relevance of GM volume deficits in chronic schizophrenia affecting frontal brain regions is also highlighted in our study by the finding of a significant negative correlation between illness duration and GM deficits in schizophrenia patients in the right dorsolateral prefrontal cortex, which remained significant after accounting for the effects of age of disease onset and degree of antipsychotic exposure.

One issue when interpreting results of widespread brain volume deficits in samples of chronic schizophrenia patients is the extent to which such findings may vary specifically as a function of greater illness severity, rather than reflecting the continuation of schizophrenia over time. Cases of schizophrenia that are more severe could conceivably display, at the time of disease onset, greater brain abnormalities that may not necessarily progress over time (Zipursky et al., 2013). In our study, a difference in the recruitment strategy between first-episode and chronic schizophrenia patients likely favoured the inclusion of more severe cases of schizophrenia in the latter group. For the recruitment of the first-episode group, a team of researchers actively surveilled local mental health services in a predefined catchment area (Schaufelberger et al., 2007), thus allowing the recruitment of cases of varying degrees of severity in the schizophrenia spectrum; a follow-up investigation after a mean of 1.5 years actually revealed remission of symptoms in a significant minority of schizophrenia cases (Schaufelberger et al., 2011). Conversely, our chronic schizophrenia patients were selected from tertiary hospital settings, thus potentially biasing the recruitment towards more severe cases, some of which with a history of recurrent hospital admissions. It is therefore likely that the finding of more widespread cortical GM volume deficits in our chronic schizophrenia group was influenced by the inclusion of more severe cases in that specific group, rather than simply reflecting the overall effects of greater illness duration in the chronic schizophrenia group. This possibility is reinforced by the fact that the chronic schizophrenia group in our study had substantially earlier disease onset relative to the first-episode group, since it is well known that earlier age of onset of schizophrenia is associated with worse outcome (Hafner and Nowotny, 1995, Clemmensen et al., 2012). Accordingly, our voxelwise search for correlations between regional GM volumes and clinical moderators indicated the presence of significant positive correlations between age of disease onset and GM volumes in several of the brain regions where chronic schizophrenia patients displayed reduced GM volumes in comparison to both first-episode patients and healthy controls.

The notion that greater GM volume deficits in schizophrenia depends on clinical severity rather than simply reflecting illness chronicity is also supported by a number of recent longitudinal MRI studies of schizophrenia which have suggested that significant progression of brain volume deficits in schizophrenia over time relative to controls may be present predominantly in a subset of patients with more severe illness and worse prognosis (Vita et al., 2012, van Haren et al., 2011). Included among those MRI investigations is a study in which a subset of the present first-episode schizophrenia sample was re-scanned after a mean of 5 years, with findings of GM decrements over time being detected in selected cortical regions only in schizophrenia subjects with a more severe, non-remitting course of illness (Rosa et al., 2015). However, in this study, we did not find any significant correlations between regional brain volumes and overall illness severity, suggesting that such moderator may be part of a complex interaction of factors with different weights on the brain structural abnormalities detectable in chronic schizophrenia.

### 4.3 Relationship between brain volume deficits and antipsychotic use

The continued use of antipsychotic medication has been previously highlighted as a potentially contributing factor influencing on the progression of GM volume deficits in chronic schizophrenia patients (Fusar-Poli et al., 2013, Torres et al., 2013, Veijola et al., 2014, Vita et al., 2015, Guo et al., 2015). This possibility has been supported by studies in animals showing structural brain changes in association with chronic antipsychotic administration (Dorph-Petersen et al., 2005, Konopaske et al., 2007, Konopaske et al., 2008). Recently, a meta-analysis of longitudinal MRI studies found progressive GM decreases among schizophrenia patients that were inversely correlated with cumulative exposure to antipsychotics, and such relationship could not be accounted for by illness duration or severity (Fusar-Poli et al., 2013). One other meta-analysis of longitudinal MRI studies focusing on typicality of antipsychotics found a significant correlation between cumulative antipsychotic exposure and progressive GM deficits in schizophrenia, although such changes were less prominent among patients treated with second-generation antipsychotics (Vita et al., 2015).

In contrast with the previous multisite VBM-based mega-analyses of schizophrenia (Gupta et al., 2014, Segall et al., 2009), we were able to carefully quantify the lifetime cumulative dosage of antipsychotics in our schizophrenia patients. With that strategy, we did find significant negative correlations between life-time cumulative exposure to antipsychotics and global brain volumes in the schizophrenia group, after accounting for the effects duration of illness. Such results are consistent with findings of previous animal studies which have demonstrated diffuse reductions in brain volume and weight in macaque monkeys exposed to olanzapine or haloperidol compared to placebo (Dorph-Petersen et al., 2005) as well as meta-analytic data from longitudinal MRI studies in schizophrenia patients showing that global GM volume decrements over time are related to cumulative exposure to antipsychotic treatments (Fusar-Poli et al., 2013). Thus our results gives support to the hypothesis that the continued use of antipsychotics may significantly influence the progression of neuroanatomical deficits in schizophrenia (Zipursky et al., 2013).

It should be noted that we found no significant correlations between indices of antipsychotic exposure and relative regional GM volume deficits in schizophrenia patients. Therefore, we cannot attribute the findings of regional GM volume deficits located in fronto-temporal cortices in chronic schizophrenia subjects to the continued exposure to antipsychotic drugs (Fusar-Poli et al., 2013, Torres et al., 2013, Veijola et al., 2014, Vita et al., 2015, Guo et al., 2015). In light of the results of the meta-analysis by Vita et al. (Vita et al., 2015), the preponderance of patients using second-generation antipsychotics in our chronic sample (64% vs. 36%) may have contributed to the absence of findings relating medication use to relative GM abnormalities affecting fronto-temporal regions in the present study.

The combined findings of our study regarding medication use, with effects on global rather than regional brain volumes in schizophrenia, are pioneer for a cross-sectional VBM mega-analysis. Nevertheless, this remains an open question, yet to be scrutinized in further mega-analyses of cross-sectional and longitudinal MRI studies.

### 4.4 Strengths and limitations

Our study has several strengths. With our multisite study design, we gained statistical power by pooling together large samples of schizophrenia patients and demographically matched controls. The potentially confounding effects of heterogeneous image acquisition protocols were addressed by using a uniform image preprocessing pipeline and by entering site/scanning protocol in the GLM used for the statistical analysis. Previous technical reports have demonstrated that results of analyses performed by pooling together MRI datasets from multiple centers are not substantially confounded by scanner differences when such methodological approach is applied (Stonnington et al., 2008, Pardoe et al., 2008). Similar strategies have been employed in two previous mega-analyses of MRI datasets from schizophrenia patients and controls (Gupta et al., 2014, Segall et al., 2009). Also, we conducted repeated analyses of regional GM volumes with covariance for different indices of global brain volumes, which varied in regard to the involvement of the distinct tissue compartments in the brain; the consistency of the results obtained in all analyses ensured that our regional GM volume findings were not influenced by global volume differences variably affecting different brain compartments in schizophrenia patients and controls. Finally, in contrast to the other multisite VBM studies of schizophrenia published to date, which gathered subjects from a wide range of geographical areas across USA and Europe (Gupta et al., 2014, Segall et al., 2009), our participants were drawn from only two urban centers separated by approximately 195 miles in the state of São Paulo, Brazil. Thus we potentially reduced variability of brain measurements related to geographical and environmental differences across separate MRI scanning sites (Kirkbride et al., 2012).

On the other hand, we acknowledge that our study is not without limitations. First, while attempting to assess the influence of various clinical moderators on GM volumes, we were unable to address the role of some other potentially relevant confounders evaluated in the previous neuroimaging literature on schizophrenia, such as changes in patterns of antipsychotic usage, treatment adherence, duration of untreated psychosis and substance abuse (van Haren et al., 2008). Also, we cannot rule out in our study the influence of other confounders that vary systematically between groups of patients with psychiatric disorders and controls, such as smoking habits, other cardiovascular risk factors and metabolic comorbidities (Weinberger & Radulescu, 2015). Finally, it could be argued that the 1.5 T magnetic field strength of all MRI scanners that provided data for this study could limit the results of our analyses. Although increasing the magnetic field strength from 1.5 to 3 T theoretically would roughly double the signal-to-noise ratio, with consequent higher contrast-to-noise to better differentiate GM and other tissues (Ho et al., 2010), conversely 3 T magnetic resonance images often have an increased level of artifact (Ho et al., 2010, Bernstein et al., 2006). The latter aspect potentially affects the accuracy of automated algorithms that classify tissue into gray and white matter components (Ho et al., 2010, Sled et al., 1998) as well as leading to local spatial distortion and artifactual local variations in image intensity (Ho et al., 2010). Moreover, recent VBM (Marchewka et al., 2014, Tardif et al., 2010) and other structural MRI studies that used other image processing methods (Ho et al., 2010) have found no or little evidence of an impact of field strength on the results of analyses investigating brain abnormalities in patients with brain disorders. Finally, recent VBM multisite mega-analyses in schizophrenia were carried out using data acquired with different scanners at 1.5 T, 3 T and 4 T and this factor has not been found to limit results and conclusions (i.e., in a subset of subjects scanned in two separate sites using different acquisition protocols, there was no significant interaction between the effects of scanner and group) (Segall et al., 2009).

### 4.5 Conclusion

In conclusion, the results of this multi-site VBM study of schizophrenia carried out in Brazil reinforce the notion that structural brain changes associated with the diagnosis of schizophrenia are more widespread in chronic schizophrenia compared to first-episode patients, mainly affecting fronto-temporal regions and the insula. Our VBM findings of significant correlations also suggest that relative GM volume deficits may be greater in (presumably more severe) cases with earlier age of onset, as well as varying as a function of illness duration in specific frontal brain regions. Finally, our results highlight the potentially complex effects of the continued use of antipsychotic drugs on structural brain abnormalities in schizophrenia, as we found that cumulative doses of antipsychotics affected brain volumes globally rather than selectively on frontal-temporal regions.

## Funding

U.S.T. was supported by a PhD grant from Foundation for the Support of Research in the State of São Paulo (Fundação de Amparo à Pesquisa do Estado de São Paulo, FAPESP), Brazil (process 11/18631-1). JASC is partly funded by a Conselho Nacional de Desenvolvimento Científico e Tecnológico (CNPq - Brazil) Productivity fellowship. G.F.B. is partly funded by CNPq, Brazil. The funders had no role in study design, data collection and analysis, decision to publish, or preparation of the manuscript.

## Figures and Tables

**Table 1:** Scanning Sequences Used in Each Magnetic Resonance Imaging Study.

Study	Author (year)	N			Type of 1.5 T MRI scanner		MRI scan sequence parameters							
Patients	Controls	Total	Vendor	Model	Sequence	TR (ms)	TE (ms)	FA	Orientation	Thickness (mm)	Field of view	Matrix size
1	Sallet et al. (2003)	35	15	50	Philips	Gyroscan S15-ACS	FFE	30	9	30°	Coronal	1.2	240	256 × 256
2	de Souza Crippa et al. (2006)	20	16	36	Siemens	Magneton Vision	FLASH	9.7	4	12°	Sagittal	1.0	256	256 × 256
3	Schaufelberger et al. (2007)	62	94	156	General Electric	Signa Horizon	SPGR	21.7	5.2	20°	Axial	1.5	220	256 × 192
4	Bassitt et al. (2007)	44	26	70	General Electric	Signa Horizon	SPGR	9	1.9	20°	Sagittal	1.2	220	256 × 192

TR = repetition time; TE = echo time, FA = flip angle.

**Table 2:** Demographic and clinical characteristics of schizophrenia patients and controls.

Characteristic	Schizophrenia patients			Statistical analysis^a^			Controls (n = 151)	Statistical analysis^b^		
Chronic (n = 99)	First-Episode (n = 62)	Total (n = 161)	*t* or χ^2^	df	p	*t* or χ^2^	df	p
Gender, n (%)										
Male	67 (67.7)	44 (31.0)	111 (68.9)	0.19	1	0.728	87 (57.6)	4.31^a^	1	0.038
Female	32 (32.3)	18 (69.0)	50 (31.1)	64 (42.4)
Age, years: mean ± s.d.	32.1 ± 8.0	27.7 ± 8.1	30.4 ± 8.3	3.42	129.45	0.001	30.6 ± 9.0	− 0.18	304.15	0.857
Education, years: mean ± s.d.	10.1 ± 3.1	8.6 ± 3.8	9.5 ± 3.5	2.47	110.17	0.01	10.7 ± 4.7	− 2.57	310	0.011
Age of onset, years: mean ± s.d.	20.2 ± 5.2	26.8 ± 8.1	22.7 ± 7.2	− 6.21	159	< 0.0001				
Duration of illness, years: median ± s.d.	11.0 ± 8.2	0.48 ± 1.2	5.0 ± 8.5	10.7	159	< 0.0001
PANSS score, total^c^: mean ± s.d.	62.6 ± 18.4^c^	47.8 ± 11.9	56.1 ± 17.4^c^	5.48	139	< 0.0001

Antipsychotics										
Typical, n (%)	15 (15.2)	43 (69.4)	58 (36.0)	48.6	1	< 0.0001				
Atypical, n (%)	84 (84.8)	19 (30.6)	103 (64.0)
Estimated life-time cumulative exposure, dose-years^d^: mean ± s.d.	4331.5 ± 4988.2	162.4 ± 568.5	2726.0 ± 4416.5	6.55	159	< 0.0001

PANSS = Positive and Negative Syndrome Scale.

a Chronic vs. first-episode schizophrenia patients.

b Total schizophrenia patients group vs. controls; continuous variables were analyzed through the *t* test and categorical variables through Pearson's χ^2^ test.

c Data not available for one study (de Souza Crippa et al., 2006).

d Estimated life-time cumulative antipsychotics exposure expressed in dose-years after transformation in mg of chlorpromazine equivalents (Andreasen et al., 2010).

**Table 3:** Comparison of global brain volumes between schizophrenia patients and controls.

Global volumes	Schizophrenia patients			Statistical analysis^a^			Controls (n = 151)	Statistical analysis^b^		
Chronic (n = 99)	First-episode (n = 62)	Total (n = 161)	*t*	df	p	*t*	df	p
Global GM volume (mm^3^): mean ± s.d.	667.71 ± 65.6	671.39 ± 79.2	669.13 ± 70.9	− 0.32	159	0.750	661.94 ± 70.2	0.90	310	0.369
Global WM volume (mm^3^): mean ± s.d.	507.21 ± 55.3	520.10 ± 64.1	512.17 ± 59.0	− 1.31	115.56	0.194	509.77 ± 57.7	0.36	310	0.717
Global CSF volume (mm^3^): mean ± s.d.	301.05 ± 36.9	275.55 ± 37.1	291.23 ± 38.9	4.25	159	< 0.0001⁎	276.79 ± 41.9	3.14	310	0.002
TBV (mm^3^): mean ± s.d.	1174.93 ± 117.7	1191.49 ± 141.8	1181.31 ± 127.3	− 0.80	159	0.424	1171.71 ± 124.7	0.67	309	0.502
TIV (mm^3^): mean ± s.d.	1475.98 ± 146.6	1467.05 ± 174.0	1472.54 ± 157.3	0.35	159	0.727	1448.51 ± 158.0	1.34	308	0.180

GM = gray matter; WM = white matter; CSF = cerebrospinal fluid; TBV = total brain volume; TIV = total intracranial volume.

a Chronic vs. first-episode schizophrenia patients.

b Total schizophrenia patients group vs. controls. Continuous variables were analyzed through the *t* test.

⁎ This result did not remain statistically significant when analysis was corrected for the effect of age of onset (p = 0.590).

**Table 4:** Regional gray matter volume reductions schizophrenia patients (chronic + first-episode groups) relative to controls based on voxel-based morphometry analyses.

Anatomical region	Number of voxels in each cluster	Peak Z score^a^	Co-ordinates of voxel of maximal statistical significance (MNI space)
Whole brain analysis (FWE-corrected at p < 0.05)			
Left insula, left inferior frontal cortex	706	6.03	− 35, 23, 1
Right insula, right striatum	104	5.09	45, 11, − 6

Additional findings based on small volume-corrected analyses (FWE-corrected at p < 0.05)			
Right dorsolateral prefrontal cortex	1051	4.23	21,59, 6
Left dorsolateral prefrontal cortex	643	5.96	− 38, 35, 1
Right anterior cingulate cortex	165	3.66	2, 51, 13
Left anterior cingulate cortex	61	4.46	− 3,53, 12
Right thalamus	179	4.03	2, − 13, 6
Left thalamus	90	3.94	0, − 13, 6
Left hippocampus/parahippocampal gyrus	120	3.75	− 30, − 30, 15

MNI = Montreal Neurological Institute; FWE = family-wise error.

a Z score for the voxel of maximal statistical significance in each cluster.

**Table 5:** Regional gray matter volume reductions in first-episode schizophrenia patients relative to controls based on voxel-based morphometry analyses.

Anatomical region	Number of voxels in each cluster	Peak Z score^a^	Co-ordinates of voxel of maximal statistical significance (MNI space)
Findings based on small volume-corrected analyses (FWE-corrected at p < 0.05)			
Right striatum	49	3.67	27, 5, − 11
Right hippocampus/parahippocampal gyrus	199	4.23	33, − 34, − 17
Right amygdala	84	4.04	27, 5, − 15
Right insula	28	3.55	42, 11, − 14
Left insula	29	3.64	− 35, 15, − 17

MNI = Montreal Neurological Institute; FWE = family-wise error.

a Z score for the voxel of maximal statistical significance in each cluster.

**Table 6:** Regional gray matter volume reductions in chronic schizophrenia patients relative to controls based on voxel-based morphometry analyses.

Anatomical region	Number of voxels in each cluster	Peak Z score^a^	Co-ordinates of voxel of maximal statistical significance (MNI space)
Whole brain analysis (FWE-corrected at p < 0.05)			
Bilateral hemisphere cluster, including: bilateral superior and orbital frontal cortices, right middle frontal cortex, bilateral anterior cingulate cortices	8616	7.01	11, 47, 10
Left insula, left inferior frontal cortex	1203	6.43	− 38, 35, 1
Right insula, right inferior frontal cortex	510	6.19	44, 18, − 5
Right superior and middle temporal cortices	1496	5.84	53, − 24, − 5
Left postcentral gyrus	49	5.54	− 41, − 24, 43
Left fusiform gyrus	77	5.22	− 36, − 43, − 14
Right thalamus	25	5.19	11, − 10, 3
Right supramarginal gyrus	46	5.01	46, − 45, 42
Left precentral gyrus	20	4.92	− 48, − 3, 37
Right calcarine gyrus	28	4.88	11, − 63, 10
Left median cingulate and paracingulate gyrus	22	4.87	− 9, − 7, 48
Left hippocampus/parahippocampal gyrus	27	4.78	− 27, − 34, − 12

Additional findings based on small volume-corrected analyses (FWE-corrected at p < 0.05)			
Left thalamus	129	6.43	− 9, − 7, 3
Right hippocampus/parahippocampal gyrus	69	5.19	14, − 6, − 20
Right temporal superior gyrus	898	4.26	53, − 24, − 5

MNI = Montreal Neurological Institute; FWE = family-wise error.

a Z score for the voxel of maximal statistical significance in each cluster.

**Table 7:** Regional gray matter volume reductions in chronic schizophrenia patients relative to first-episode schizophrenia patients based on voxel-based morphometry analyses.

Anatomical region	Number of voxels in each cluster	Peak Z score^a^	Co-ordinates of voxel of maximal statistical significance (MNI space)
Whole brain analysis (FWE-corrected at p < 0.05)			
Left superior temporal cortex, extending to left precentral and postcentral cortices	2642	7.42	− 41, − 25, 6
Right superior and middle temporal cortices, extending to right supramarginal and angular gyri	5830	7.22	46, − 46, 48
Left middle temporal cortex, extending to left angular gyrus	841	7.15	− 44, − 64, 22
Left superior and inferior parietal cortices	665	7.06	− 29, − 48, 54
Left inferior temporal cortex, extending to left parahippocampal gyrus and left inferior occipital, fusiform and lingual cortices	1508	6.79	− 36, − 52, − 3
Left precentral cortex	245	6.42	− 30, − 13, 45
Left postcentral cortex	256	6.26	− 33, − 31, 54
Left inferior frontal cortex	294	5.89	− 29, 33, − 3
Left insula	108	5.12	− 41, 5, 12

Additional findings based on small volume-corrected analyses (FWE-corrected at p < 0.05)			
Right thalamus	905	6.16	11, − 10, 3
Right amygdala	330	5.42	− 6, 6, − 3

MNI = Montreal Neurological Institute; FWE = family-wise error.

a Z score for the voxel of maximal statistical significance in each cluster.

**Table 8:** Regional gray matter volume reductions in chronic schizophrenia patients relative to first-episode schizophrenia patients based on voxel-based morphometry analyses after correction for the confounding effect of age of onset.

Anatomical region	Number of voxels in each cluster	Peak Z score^a^	Co-ordinates of voxel of maximal statistical significance (MNI space)
Whole brain analysis (FWE-corrected at p < 0.05)			
Left precentral cortex	238	7.09	− 48, − 4, 40
Right calcarine, precuneus, cuneus and lingual cortices	1166	6.95	12, − 63, 13
Right superior and middle temporal cortices	3206	6.76	45, − 4, − 24
Left superior temporal cortex	1228	6.45	− 41, − 27, 6
Right inferior parietal cortex, extending to right supramarginal and angular gyri	552	6.27	46, − 46, 48
Left middle temporal cortex	367	6.18	− 44, − 64, 22
Left superior and inferior parietal cortices	163	6.16	− 27, − 48, 54
Right thalamus	54	5.88	11, − 10, 3
Left postcentral cortex	45	6.26	− 44, − 24, 46
Right superior frontal cortex	177	5.50	9, 39, 46
Right inferior frontal cortex	33	5.24	33, 30, − 11

Additional findings based on small volume-corrected analyses (FWE-corrected at p < 0.05)			
Right anterior cingulate cortex	2629	6.72	17, 44, 19
Left anterior cingulate cortex	2131	7.42	− 5, 51, − 2
Left striatum	323	5.22	− 6, 6, − 3
Right hippocampus/parahippocampal gyrus	319	6.48	42, − 16, − 14
Left hippocampus/parahippocampal gyrus	485	5.58	− 24, − 39, − 8
Right insula	274	4.33	45, 18, − 5
Left insula	298	3.90	− 36, 0, 6
Left thalamus	181	5.70	− 9, − 6, 4

MNI = Montreal Neurological Institute; FWE = family-wise error.

a Z score for the voxel of maximal statistical significance in each cluster.

**Table 9:** Results of small volume-corrected analyses (FWE-corrected at p < 0.05) showing positive correlations between gray matter regional volumes and age of onset in the conjoined groups.

Anatomical region	Number of voxels in each cluster	Peak Z score^a^	Co-ordinates of voxel of maximal statistical significance (MNI space)
All patients^b^			
Right dorsolateral prefrontal cortex	740	5.19	42, 15, 15
Left dorsolateral prefrontal cortex	221	4.30	− 32, 29, 12
Right anterior cingulate cortex	475	3.87	17, 42, 21
Left anterior cingulate cortex	174	3.69	− 2, 53, 3
Right insula	108	4.16	46, 12, 3
Left insula	1190	4.31	− 30, 29, 12
Right hippocampus/parahippocampal gyrus	286	4.07	36, − 34, − 5
Right superior temporal gyrus	395	4.23	60, − 48, 22

Conjunction of three groups with available PANSS total scores^c^			
Right dorsolateral prefrontal cortex	204	4.34	18, 42, 24
Left dorsolateral prefrontal cortex	106	3.90	− 32, 29, 12
Left anterior cingulate cortex	103	3.51	− 2, 54, − 2
Left insula	189	3.80	− 30, 29, 12
Right hippocampus/parahippocampal gyrus	252	3.77	36, − 34, − 3
Right superior temporal gyrus	451	4.37	60, − 49, 22

First-episode^d^			
Right superior temporal gyrus	21	3.78	42, − 31, 4

MNI = Montreal Neurological Institute; FWE = family-wise error.

a Z score for the voxel of maximal statistical significance in each cluster.

b After accounting for effects of lifetime cumulative exposure to antipsychotics.

c After accounting for effects of lifetime cumulative exposure to antipsychotics and overall illness severity.

d After accounting for effects of lifetime cumulative exposure to antipsychotics and duration of disease.

**Table 10:** Results of small volume-corrected analyses (FWE-corrected at p < 0.05) showing negative correlations between gray matter regional volumes and duration of disease in the conjoined and first-episode groups.

Anatomical region	Number of voxels in each cluster	Peak Z score^a^	Co-ordinates of voxel of maximal statistical significance (MNI space)
All patients^b^			
Right insula	162	4.11	48, 8, 6
Left insula	1583	4.33	− 42, 5, 10
Right dorsolateral prefrontal cortex	934	4.85	51, 15, 7
Left dorsolateral prefrontal cortex	632	4.23	− 41, 6, 10
Right hippocampus/parahippocampal gyrus	226	3.86	8, 26, 21
Left hippocampus/parahippocampal gyrus	113	3.64	− 27, − 30, − 6

Conjunction of three groups with available PANSS total scores^c^			
Right insula	62	3.60	48, 8, 6
Left insula	806	3.78	− 29, 29, − 3
Right dorsolateral prefrontal cortex	1015	4.61	18, 44, 24
Left dorsolateral prefrontal cortex	286	3.82	− 30, 35, − 2
Right hippocampus/parahippocampal gyrus	123	4.19	29, − 19, − 9
Left hippocampus/parahippocampal gyrus	190	3.77	− 27, − 34, − 9
Right anterior cingulate cortex	23	4.07	17, 45, 21
Left anterior cingulate cortex	112	3.91	− 3, 53, 1

Conjunction of three groups with available PANSS total scores^d^			
Right dorsolateral prefrontal cortex	258	3.94	28, 15, 52

First-episode^d^			
Left superior temporal gyrus	108	3.82	− 54, − 24, 10

MNI = Montreal Neurological Institute; FWE = family-wise error.

a Z score for the voxel of maximal statistical significance in each cluster.

b After accounting for effects of lifetime cumulative exposure to antipsychotics.

c After accounting for effects of lifetime cumulative exposure to antipsychotics and overall illness severity.

d After accounting for effects of lifetime cumulative exposure to antipsychotics, overall illness severity and age of onset.
